# Bronchopleurocutaneous fistula in absence of empyema: A rare presentation of pulmonary tuberculosis

**DOI:** 10.4103/0970-2113.76310

**Published:** 2011

**Authors:** M. Azfar Siddiqui, Mohammad Shameem, Jamal Akhtar, Ummul Baneen, Rakesh Bhargava, Zuber Ahmed, Prakhar Sharma, Nafees Ahmad Khan

**Affiliations:** *Department of Radiology, Jawaharlal Nehru Medical College, Aligarh, Muslim University, Aligarh, Uttar Pradesh, India*; 1 *Department of Tuberculosis and Chest Disease, Jawaharlal Nehru Medical College, Aligarh, Muslim University, Aligarh, Uttar Pradesh, India E-mail: akhtar.jamal10@gmail.com*

Sir,

A 60-years-old male presented to us with complaint of fever and cough with expectoration for last one month and pus discharging sinus on anterolateral aspect of right side chest wall for last one week. There was no history of hemoptysis, chest pain, dyspnoea or any previous swelling on chest wall at the site of discharging sinus. There was no previous history of tuberculosis or any anti-tubercular drugs (ATT) intake. Also there was no past history of intercostal tube drain (ICD) insertion or any history suggestive of pleural effusion. On respiratory system examination, crepts were present in bilateral lung fields. Examination of cardiovascular system and gastrointestinal system was absolutely normal.

**Figure 1:**
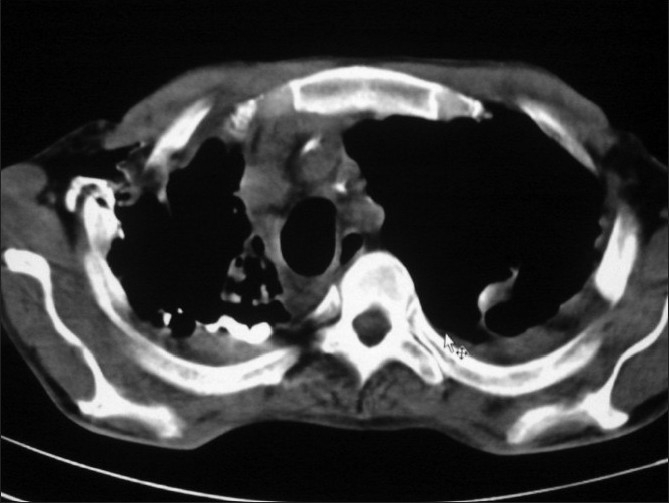
Axial chest CT demonstrates movement of dye injected in subcutaneous fistula along posterior pleural space into cavity in posterior segment of right upper lobe. From the cavity dye entered in draining segmental bronchus thus confirming the presence of bronchopleurocutaneous fistula

In laboratory investigation, ESR was raised while complete blood count (CBC), renal function test, liver function test were within normal limit. Smear and culture of sputum was positive for acid fast bacilli. Pus from discharging sinus failed to reveal any growth of pyogenic or fungal organism on staining as well as culture. CECT of -thorax was performed after injecting dye in the discharging sinus which revealed the presence of bronchopleurocutaneous fistula. Frontal CT scout view demonstrates extensive fibrocavitatory changes in both lungs more on right side with right sided mediastinal shift. It also revealed volume loss of right lung associated with extensive pleural thickening and blunting of CPA. Infant feeding tube was used for injecting dye in fistula. Axial chest CT demonstrates movement of dye injected in subcutaneous fistula along posterior pleural space into cavity in posterior segment of right upper lobe. From the cavity dye entered in draining segmental bronchus, thus confirming the presence of bronchopleurocutaneous fistula [Figure 1]. There was no evidence of empyema or any rib involvement on CECT of-thorax.

Patient was diagnosed as a case of pulmonary tuberculosis with bronchopleurocutaneous fistula. Patient was put on antitubercular drugs. Patient responded well and the fistula healed.

Bronchopleurocutaneous fistula is a pathological communication between bronchus, pleural space and skin. It usually develops due to pulmonary operations, perforating chest trauma, empyema, lung abscess, pneumonia or massive pulmonary infarction.[1–3] Occasional cases of bronchopleurocutaneous fistula associated with histoplasma and aspergillus are reported in literature,[4,5] but in these cases empyema was also present. In our patient for the first time Mycobacterium tuberculosis was implicated in the development of bronchopleurocutaneous fistula in absence of empyema. This is also the first case of pulmonary tuberculosis in our best knowledge in which patient presented with bronchopleurocutaneous fistula without having empyema. The most common presenting features of pulmonary tuberculosis are productive cough with fever, weight loss, night sweats, anorexia and chest pain. The radiological presentations are infiltrates, opacities, consolidation, cavitation, miliary lesions, hilar lymphadenopathy, bronchiectasis, collapse, pneumothorax, hydropneumothorax, effusion or empyema.[6] Bronchopleurocutaneous generally develops as a complication of empyema neccisstans. Diagnosis is made by bronchogram, fistulogram or by percutaneous fibrooptic bronchoscopy.[3]
